# Bioactive Compounds in the Ethanol Extract of Marine Sponge *Stylissa carteri* Demonstrates Potential Anti-Cancer Activity in Breast Cancer Cells

**DOI:** 10.31557/APJCP.2019.20.4.1199

**Published:** 2019

**Authors:** Muhammad Hasan Bashari, Fathul Huda, Tamia S Tartila, Sarah Shabrina, Tenny Putri, Nurul Qomarilla, Harold Atmaja, Beginer Subhan, Ikhwan Resmala Sudji, Edy Meiyanto

**Affiliations:** 1 *Department of Biomedical Sciences, Division of Pharmacology and Therapy,*; 2 *Oncology and Stem Cell Working Group, *; 3 *Department of Biomedical Sciences, Division of Physiology, *; 4 *Undergraduate Program,*; 5 *Laboratory of Advanced Biomedicine, Faculty of Medicine, Universitas Padjadjaran, Bandung,*; 6 *Department of Marine Science and Technology, Faculty of Fisheries and Marine Sciences, Bogor Agricultural University, Bogor, *; 7 *Laboratory of Biomedicine, Faculty of Medicine, Universitas Andalas, Padang, *; 8 *Cancer Chemoprevention Research Center, Faculty of Pharmacy, Universitas Gadjah Mada, Yogyakarta, Indonesia. *

**Keywords:** Anti-cancer activity- breast cancer- marine sponge- *Stylissa carteri*

## Abstract

**Objective:**

Despite advanced treatment options available, drug resistance develops in breast cancer (BC) patients requiring novel effective drugs. *Stylissa carteri*, a marine sponge predominantly living in Indonesia territories, has not been extensively studied as anti-cancer. Therefore, this study targeted to assess the anti-tumor activity of the ethanol extract of *S. carteri* in BC cells.

**Methods:**

*S. carteri* was collected from Pramuka Island, at Kepulauan Seribu National Park, Jakarta, Indonesia and extracted using ethanol. Different BC cells including MDA MB 231, MDA MB 468, SKBR3, HCC-1954 and MCF-7 cells were treated with this extract for cytotoxic analysis using MTT assay. Spheroid growth assay and apoptosis assay were conducted in HCC-1954 cells. In addition, cell migration analysis and synergistic activity with doxorubicin or paclitaxel were conducted in MDA MB 231 cells. This extract was subjected also for GC-MS analysis.

**Results:**

The results show that ethanol extract of *S. carteri* demonstrated a cytotoxic activity in BC cells. The IC_50_ of this extract was lower 15 μg/ml in MDA MB 231, MDA MB 468, SKBR3, and HCC-1954 cells. Moreover, this extract inhibited spheroids growth and induced apoptosis in HCC-1954 cells. It inhibited cell migration and demonstrated a synergistic activity with doxorubicin or paclitaxel on triggering cell death in MDA MB 231 cells. Furthermore, GC-MS analysis indicated that this extract contained 1,2-Benzenediol, Dibutyl phthalate and 9,12-Octadecadienoic acid, ethyl ester.

**Conclusion:**

Our preliminary data indicate a potential anti-tumor activity of ethanol extract of *S. carteri* in breast cancer cells.

## Introduction

Breast cancer (BC) is the first most diagnosed cancer in women worldwide, and they are becoming first main cause of cancer-related deaths in women, respectively (Torre et al., 2015). The high prevalence and mortality by BC along with weaknesses of existing managements and prevention raise the urgency and need for discovery of novel drugs (Aungsumart et al., 2007; Torre et al., 2015). 

Despite advanced BC treatment modalities are available, advanced stage of BC patients, develop resistant to current therapeutic options. Chemotherapy resistance leads into cancer progression and metastasis, thereby it remains as the greatest challenges in cancer management and responsible to cancer-related death (Hammond et al., 2016). Therefore, it is urgent to discover a novel drug for BC to overcome the resistance and provide better treatment response, which will lead in improving the survival of patients with BC.

Marine sponges have been explored worldwide for novel anti-cancer agents. One of successfully active compound originated from marine sponge is Eribulin, which has been approved by American Food and Drug Administration (FDA) for the advanced stage of triple negative BC (Candida et al., 2012). Moreover, a novel promising compound, Leiodermatolide, was just synthesized based on an isolated compound from deep marine sponges, Leiodermatium sp. from Florida. This compound demonstrates a powerful anti-tumor activity compared to paclitaxel in prostate cancer cells (Guzmán et al., 2016). Above data indicating that sponges are very potential to be studied for novel anti-cancer drug discovery. 

Indonesia as an archipelago country possesses botanical biodiversity potential that incompletely developed for novel cancer treatment. Here we evaluate *S. carteri* for its anti-tumor activities. *S. carteri* are widespread in Indo-Pacific region, including the red sea, Australian sea and many Indonesian territories (Erpenbeck et al., 2017). To date studies subjected this species for its anti-tumor activities are very limited. Nevertheless, a recent study shows that an isolated compound from *S. carteri* induces cervical cancer Hela cells death (Dewi, 2017). This study wanted to identify anti-tumor activities of ethanol extract of marine sponge *S. carteri* in different BC cells. 

## Materials and Methods

*Chemicals and reagents*

RPMI 1640 medium (cat No. 11875093), Dulbecco’s modified Eagle’s medium (DMEM) (cat no 11965-092), fetal bovine serum (FBS) (cat No. 10270106), non-essential amino acid and penicillin streptomycin (cat No. 15140122) were purchased from Gibco, USA. Dimethyl sulfoxide (DMSO) (cat No. D8418), and 3-[4,5-dimethylthiazol-2-yl]-2,5 diphenyl tetrazolium bromide (MTT) (cat No. M2128), were purchased from Sigma-Aldrich, USA. Doxorubicin and Paclitaxel were provided by Pharmacy of Hasan Sadikin Hospital, Bandung, Indonesia. All the other chemicals were of analytical grade purchased from Merck, USA.

**Figure 1:**
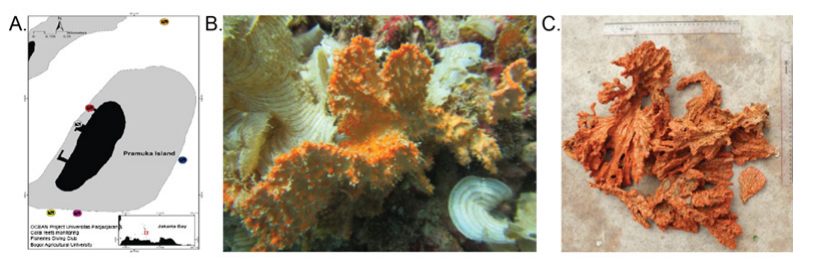
*Stylissa Carteri* was Taken from Different Sites in Pramuka Island. (A) Collected sites of *Stylissa carteri*. *Stylissa carteri *was on coral (B) and after was taken (C).

**Figure 2:**
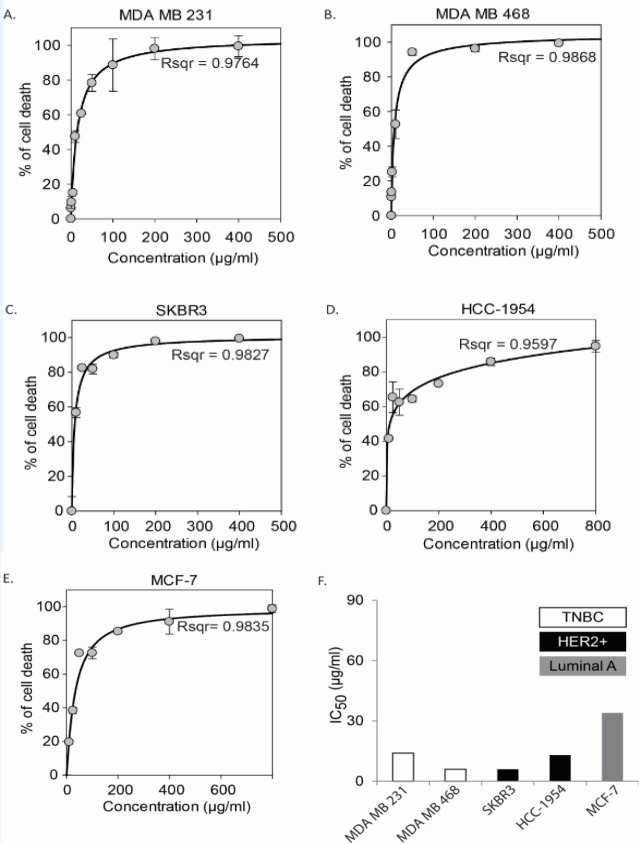
The Et (OH) Extract of *S. Carteri* Triggers Cell Death in BC Cell Lines in Dose Dependent Manner. BC cell lines were treated with Et(OH) extract for 72 hours followed by cytotoxic analysis using MTT assay. Medium with 1% DMSO was used as control. Data were presented as mean and SD from triplicate data. Drug curves (A-E) were created and IC_50_ of each cell lines (F) were analyzed using four parametric logistic model by Sigmaplot ver.12

**Figure 3:**
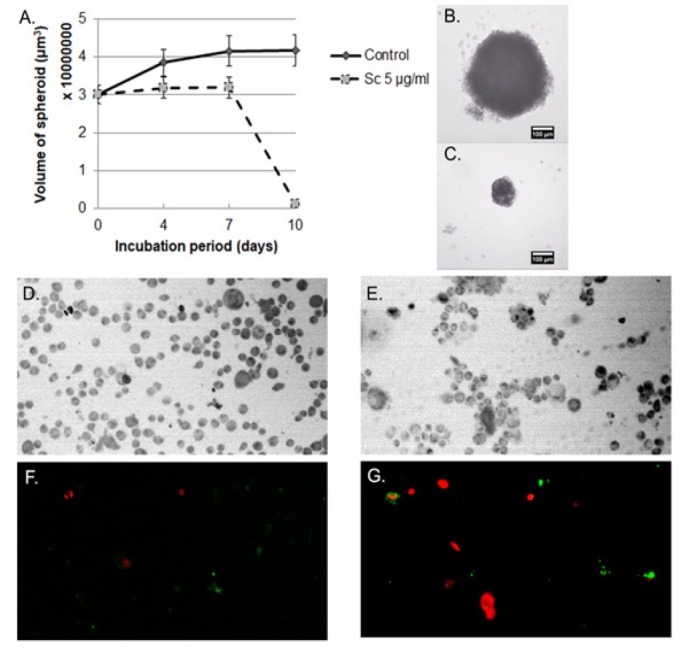
The Et (OH) extract of *S. carteri* Inhibits Spheroid Growth and Induces Apoptosis of HCC-1954 Cells. After spheroids of HCC-1954 were generated, they were treated and untreated with 5μg/ml of the Et(OH) extract of *S. carteri.* (A) Spheroid volumes were declined upon treatment. Spheroid mass on day 10th represented of control (B) and treated group (C). Data was presented as mean and SD of 7 replicates each group. HCC-1954 cells were un-treated (D, F) and treated (E,G) with 5μg/ml of the Et(OH) extract of *S. carteri* for 48 hours followed by Annexin V (green)/PI (red) staining and captured under Olympus Fluorescence microscope with 100x magnification. D, E=bright field; F, G= Fluorescence

**Figure 4:**
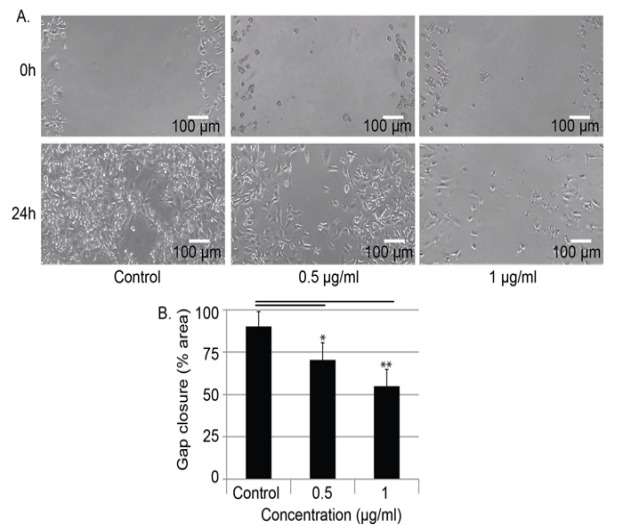
The Et (OH) Extract of *S. carteri* Inhibits Cell Migration of MDA MB 231 in Dose Dependent Manner. MDA MB 231 cells were scratched prior treated with indicated concentration of the Et(OH) extract for 24 hours. Cell gaps were capture. Medium with 1% DMSO was used as control. Data were presented as mean and SD from triplicate data. *p < 0.05; ** p < 0.01 to control

**Figure 5:**
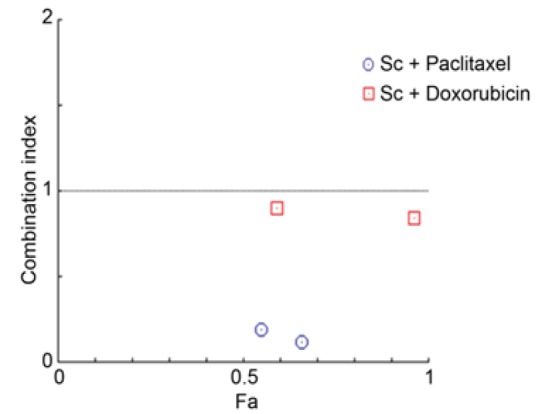
Combination of the Et (OH) Extract of *S. carteri* with Conventional BC Chemotherapy Agents Induces Synergism in TNBC Cells. MDA MB 231 cells were treated Et(OH) extract of* S. carteri *(Sc) 2 μg/ml or 10 μg/ml in combination with paclitaxel 1 or 4 nM and doxorubicin 1 or 4 nM for 72 hours prior cytotoxic assay using MTT assay. Combination index was analyzed using Compusyn Software

**Figure 6:**
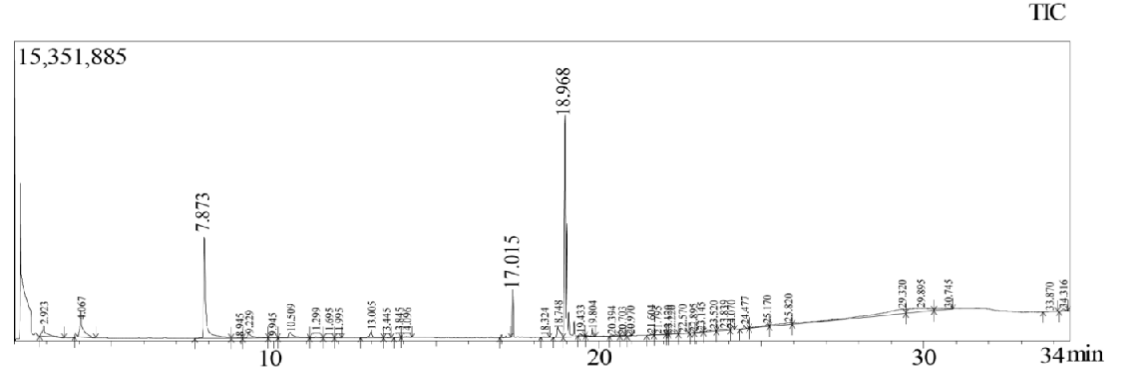
GC-MS Chromatogram of the Ethanol Extract of *S. carteri*

**Table 1:** Compounds Detected in the Ethanol Extract of *Stylissa carteri*

S.No	RT	Peak %	Name	Molecular formula
1	2.923	2.34	Propane, 1,1-diethoxy-2-methyl	C_8_H_18_O_2_
2	4.067	6.05	Butane, 1,1-diethoxy-3-methyl	C_9_H_20_O_2_
3	7.873	23.9	1,2-Benzenediol	C_6_H_6_O_2_
4	8.945	0.22	Phenol, 4-ethyl-2-methoxy	C_9_H_12_O_2_
5	9.229	1.06	1,2-Benzenediol, 4-methyl	C_7_H_8_O_2_
6	9.945	0.21	Phenol, 2,6-dimethoxy	C_8_H_10_O_3_
7	10.509	1.31	1,3-Benzenediol, 4-ethyl	C_8_H_10_O_2_
8	11.299	0.24	Phenol, 4-ethyl-2-methoxy	C_9_H12O_2_
9	11.695	0.13	Cyclooctene, 3-methyl	C_9_H_16_
10	11.995	0.15	Naphthalene, 1,2,3,4,4a,5,6,8a-octahydro-4a,8	C_15_H_24_
11	13.005	1.62	Diethyl Phthalate	C_12_H_14_O_4_
12	13.445	0.11	7-Benzofuranol, 2,3-dihydro-2,2-dimethyl	C_10_H_12_O_2_
13	13.845	0.12	Esculetin	C_9_H_6_O_4_
14	14.096	0.16	3,6-Dioxaoctanedioic acid	C12H22O2
15	17.015	0.54	Dibutyl phthalate	C_16_H_22_O_4_
16	18.324	0.16	Linoleic acid ethyl ester	C_20_H_36_O_2_
17	18.748	2.61	cis-9,cis-12-Octadecadienoic acid	C_18_H_32_O_2_
18	18.968	49.26	9,12-Octadecadienoic acid, ethyl ester	C_20_H_36_O_2_
19	19.433	0.51	Linoleic acid ethyl ester	C_20_H_36_O_2_
20	19.804	1.47	Linoleic acid ethyl ester	C_20_H_36_O_2_
21	20.394	0.57	8,10-Hexadecadien-1-ol	C_16_H_30_O
22	20.703	0.14	Ethyl 9-hexadecenoate	C_18_H_34_O_2_
23	20.97	0.18	Pentadecanoic acid	C_19_H_38_O_2_
24	21.604	0.27	6,9-Pentadecadien-1-ol	C_15_H_28_O
25	21.795	0.06	Fatty Acid	C_24_H_44_O_4_
26	22.12	0.2	Hexadecanal	C_16_H_32_O
27	22.22	0.07	Linoleic acid ethyl ester	C_20_H_36_O_2_
28	22.57	0.12	Heptadecanoic acid	C_19_H_38_O_2_
29	22.895	0.11	Pentadecanal	C_15_H_30_O
30	23.145	0.28	Palmitic acid vinyl ester	C_18_H_34_O_2_
31	23.52	0.47	Hydroxy (palmitoyloxy)propyl-octadecenoate	C_37_H_70_O_5_
32	23.839	0.4	Hydroxy (palmitoyloxy)propyl-octadecenoate	C_37_H_70_O_5_
33	24.07	0.19	Docosanoic acid	C_24_H_48_O_2_
34	24.477	0.86	Tricyclo[20.8.0.0(7,16)]triacontane	C_30_H_52_O_2_
35	25.17	0.58	Cyclohexane,1,1-(oxydi-1-ethanediyl)bis methyl	C_18_H_34_O
36	25.82	0.41	9-Borabicyclo[3.3.1]nonane, 9-(3-pentyloxy)	C_13_H_25_BO
37	29.32	1.17	Trilinolein	C_57_H_98_O_6_
38	29.895	0.92	2-Hydroxy-3(palmitoyloxy)propyl (9)-9 octadecenoate	C_37_H_70_O_5_
39	30.745	0.25	3,7,11,15-Tetramethyl-2-hexadecen-1-ol	C_20_H_40_O
40	33.87	0.07	no hit compound	
41	34.316	0.5	E,E,Z-1,3,12-Nonadecatriene-5,14-diol	C_19_H_34_O_2_

*Extraction of Stylissa carteri*

Marine sponge *S. carteri* was taken by SCUBA diving from different sites at 10 meters depth in Pramuka Island, which constitutes the Kepulauan Seribu Marine National Park located in the north of Jakarta, Indonesia (Figure 1). Species were visually identified in the field, and confirmed at Department of Marine Science and Technology, Faculty of Fisheries and Marine Sciences, Bogor Agricultural University. Samples were cut into small size and then extracted using maceration technic in ethanol according to previous study (Hardani et al., 2018).

*Cell culture and conditions*

The triple negative (TN) BC cells (MDA MB 231, MDA MB 468), HER2+ BC cells (SKBR3, HCC-1954) and luminal A BC cells (MCF-7) were used to evaluate the anti-tumor activity of Et(OH) extract of *S. carteri*. The MDA MB 231 and MDA MB 468 cells were from Dr. Thordur Oskarsson (DKFZ, Germany), SKBR3 cells were from Prof. Andreas Trumpp (DKFZ, Germany), HCC-1954 cells were from Prof. Stefan Wiemann (DKFZ, Germany), MCF-7 cells were from Dr. Ahmad Faried and Prof. Hiroyuki Kuwano.

SKBR3 cells were cultured using DMEM supplemented with non-essential amino acid, 10% heat-inactivated FBS, 1% penicillin/streptomycin and 2 mM L-glutamine in a regular cell culture incubator that contained 21% O_2_, 5% CO_2_, 37°C. All the other cell lines were cultured using RPMI 1640 medium supplemented with 10% heat-inactivated FBS, 1%. All experiments were conducted triplicate and from 3 different experiments at Laboratory of cell culture and cytogenetic, Faculty of Medicine, Universitas Padjadjaran, Indonesia.

*Cytotoxicity assay*

To evaluate cytotoxic activity of the Et(OH) extract of *S. carteri* in BC cells, we used MTT assay, as previously described (Vallet et al., 2016). Cell lines were seeded on 98-well plate a day before untreated or treated with the Et(OH) extract as indicated concentrations then incubated for 72 hours. At the last day, MTT solution was added 4 hours before stopped by DMSO. Samples were read at 550 nm with a plate reader (Thermo Scientific® Multiscan EX, Singapore).

*Spheroid formation assay*

Single multicellular BC spheroids were generated according previous study (Bashari et al., 2016). Briefly, a number of 9,000 HCC-1954 cells were seeded on agarose-coated (Sigma Aldrich, Steinheim, Germany) 96-well plates followed by 4 days incubation for initiation of spheroid formation. Spheroids were then treated or untreated with Et(OH) extract of *Stylissa carteri*. Spheroids were captured with the microscope using the camera connected with a computer and Toupview Software (version x64, 3.7.7892) using 40x magnifications. Images were analyzed using ImageJ software to have spheroid radius. Volumes of the spheroids were calculated (V = 4/3 πr^3^).

*Apoptosis assay*

In order to identify apoptosis induced cell death triggered by the Et(OH) extract of *S. carteri*, we used Dead Cell Apoptosis Kit with Annexin V FITC and PI (Invitrogen, cat.no. V13242). After HCC-1954 cells were growth on 6-well plate, cells were treated and un-treated with the Et(OH) extract of *S. carteri* for 48 hours. Cells were harvested followed by stained with Annexin V/PI according to manufacture protocol. Cell suspension were then placed on an object glass followed by captured using Olympus fluorescence microscope BX51 using the camera connected with a computer and Toupview Software (version x64, 3.7.7892) using 100x magnifications. Images were stacked using ImageJ software.

*Combination index*

Cells were treated with different concentration of Et(OH) extract of *S. carteri* alone or in combination with doxorubicin, or paclitaxel followed by the cytotoxic assay. Combination index was analyzed with Compusyn software based on Chou Talalay method (Chou, 2010). 

*Migration Assay*

To assess the anti-cell migration of Et(OH) extract of *Stylissa carteri*, we were conducted scratch/wound healing assay in MDA MB 231 cells according to previous study (Vallet et al., 2016). After gaps were created, cells were treated or untreated with Et(OH) extract of *S. carteri* in complete medium then placed in incubator. The 0th and 24th hour of treatment were captured under the microscope which connected with a computer and Toupview Software (version x64, 3.7.7892) and saved as TIFF. The gap area was measured using MRI Wound Healing Tool macro for ImageJ software (NIH) (http://dev.mri.cnrs.fr/projects/imagejmacros/wiki/Wound_Healing_Tool). 

*GC/MS analysis*

The GC/MS analyses were carried out on Shimadzu single quadrupole GCMS-QP2010 Ultra gas chromatograph-mass spectrometer according to previous study (Vetvicka and Vetvickova, 2016). Briefly, the GC was equipped with a 30 m x 0.25 mm RP-5 non-polar column (Shimadzu) with 0.25 l m film thicknesses. The MS was run in the electron impact ionization mode with an ionizing energy of 70 eV, scanning from m/z 1 to 2,000 at 0.3scan/sec. The ion source temperature was 300°C, and the quadrupole temperature was 280°C while the electron multiplier voltage was maintained at 0.8 kV. The chromatographic conditions were identical to those used for gas chromatography analysis. Helium was used as carrier gas, the flow through the column was 1 mL/min, and the split ratio was set to 400:1. The column was maintained at 40°C for 10 min, increased to 180°C at a rate of 2.5°C/min, and finally maintained at a rate of 20 min. Injection volume of the sample was 0.2µL. For the identification of the compounds, retention times and retention index were confirmed with database from software NIST11 Mass Spectral Library.

*Statistical analysis*

Four-parametric-logistic model by Sigmaplot for windows ver.12 software (Systat Software Inc) was used to generate drug curves and to analyze IC_50_. Anova test and posthoc Holm-Sidak method were used to determine the statistical significance of differences observed in treated versus control cultures. Data was significantly different if p < 0.05.

## Results

*The Et(OH) extract of S. carteri has cytotoxic activity in BC cell lines*

Cytotoxic effects of the Et(OH) extract of *S. carteri* were evaluated using MTT assay in different BC cell lines including the TNBC cells, MDA MD 231 and MDA MB 468; HER2+ cells, SKBR3, and HCC-1954 as well as ER+ BC cells, MCF-7. Our data revealed that the Et(OH) extract of *S. carteri* induced cell death in all BC cells lines in dose dependent manner (Figure 2A-E). The IC_50_ of the Et(OH) extract of *S. carteri* were less than 90 μg/ml in all tasted BC cell lines (Figure 2F). Interestingly, the IC_50_ of the Et(OH) extract of *S. carteri* were lower in the aggressive BC subtype cells, TNBC and HER2+ than in ER+ BC cells (Figure 2F). 

*The Et(OH) extract of S. carteri inhibits spheroid growth and induces apoptosis in HCC-1954 cells*

Next, we evaluate effects of the Et(OH) extract of *S. carteri* in BC cells using 3-dimentional culture system. Spheroids of HCC-1954 were treated or untreated with the Et(OH) extract of *S. carteri*. Data showed that the Et(OH) extract of *S. carteri* induced declining of BC spheroids volumes along with incubation periods (Figure 3A-C). Importantly, the Et(OH) extract of *S. carteri* induced apoptosis in HCC-1954 cells (Figure 3D-G).

*The Et(OH) extract of S. carteri inhibits TNBC cell migration in dose dependent manner*

Migration of cancer cells is one of the key aspects of cancer metastasis. Therefore, we then wonder whether the Et(OH) extract of *S. carteri* able to inhibit BC cell migration. Utilizing a basic cell migration assay, wound healing assay, the aggressive TNBC cells, MDA MB 231 cells were treated with low concentration of Et(OH) extract of *S. carteri*. In control group, the gap in MDA MB 231 cells was closed while in the treated groups, the gaps were still opened. Both concentrations of Et(OH) extract of *S. carteri* significantly inhibited MDA MB 231 cells migration (p<0.05) (Figure 4).

*The Et(OH) extract of S. carteri induces synergistic cell death with conventional chemotherapy agents in TNBC cells*

Heretofore our data showed a promising anti-tumor activity of the Et(OH) extract of *S. carteri* by inducing cell death and inhibiting cell migration. Considering that cancer cells were activating multiple pathways for resisting from cell death, it is important to target cancer cell with multiple agents. Here we evaluated the combination effect of Et(OH) extract of *S. carteri* with paclitaxel or with doxorubicin as two of main BC chemotherapy regiment. Importantly, these combinations trigger synergistic effect on cell death of TNBC cells (Figure 5).

*GC-MS data of the Et(OH) extract of S. carteri*

The compounds detected in the Et(OH) extract of *S. carteri* were identified by GC-MS analysis (Figure 6). Based on chromatogram, it was indicated that the prominent compounds in the Et(OH) extract of *S. carteri* were 1,2-Benzenediol, Dibutyl phthalate and 9,12-Octadecadienoic acid, ethyl ester (Table 1). 

## Discussion

Marine sponges have been studied worldwide for its anti-tumor activities. However, there are very limited studies on *S. carteri* for its anti-cancer activities. Alkaloid compounds, (Z)-debromohymenialdisine dan (Z)-hymenialdisine from methanol extract of *S. carteri* reveals a cytotoxic effects in MONO-MAC-6 leukemic cells (Eder et al., 1999). Similarly, an alkaloid compounds of *S. carteri* which isolated from red sea, demonstrates cytotoxic effects in HCT-116 cells (Hamed et al., 2018). In addition, a recent study shows that the methanol crude extract of *S. carteri* has cytotoxic effects in cervical cancer HeLa cells (Dewi, 2017). Our previous data demonstrates that the Et(OH) extract of *S. carteri* triggers cell death in both parental HeLa cells and paclitaxel resistance HeLa cells (Hardani et al., 2018). 

Here our data shows a promising anti-tumor activity of the Et(OH) extract of *S. carteri* in BC cells. The Et(OH) extract of *S. carteri* prompts BC cell death in dose dependent manner (Figure 2). Importantly, the aggressive BC cell types are more sensitive to this extract (Figure 2F). Along with this finding, the Et(OH) extract of *S. carteri* degenerates spheroids and induces apoptosis in an aggressive BC cell line, HCC-1954 cells (Figure 3). Moreover, the Et(OH) extract of *S. carteri* not only inhibits the TNBC cell migration (Figure 4) but it also produces synergistic antitumor activity with the conventional anti-BC agents, doxorubicin and paclitaxel (Figure 5). 

Sponges contain different secondary metabolites that are proposed to have anti-tumor effects. Stylisin has been isolated from genus Stylissa and demonstrated antioxidant properties as well as cytotoxic effects to cancer cells (Sima and Vetvicka, 2011). In addition, previous study shows that debromohymenialdisine (DBH), hymenialdisine (HD), and oroidin, which isolated from *S. carteri* inhibits Human Immunodeficiency Virus 1 (HIV-1) (O’Rourke et al., 2016). Based on photochemistry analysis which was conducted by Central Lab Universitas Padjadjaran with register no. S-420/LS-AK.143/2017, our Et(OH) extract of *S. carteri* contains flavonoid, triterpenoid and steroid. Moreover, using GC-MS analysis our extract showed some bioactive compounds. According to its chromatogram, this extract contained 1,2-Benzenediol, Dibutyl phthalate, 9,12-Octadecadienoic acid, ethyl ester and many other compounds (Table 1). Previous studies have been shown their effects on cancer cells. Dibutyl phthalate is considered as carcinogenic agent. This compound induced cell proliferation and invasiveness in breast cancer cells (Hsieh et al., 2012). This compound also induces apoptosis in neural cells (Wójtowicz et al., 2017). Moreover, 9,12-Octadecadienoic acid, fatty acid, was also identified from other marine sponge, *Scopalina ruetzleri*, which collected from South Brazilian coastline. Ethyl acetate fraction of *S. ruetzleri *induces cell death of human glioma cells and neuroblastoma cells (Biegelmeyer et al., 2015). 

Having a promising data of the Et(OH) extract of *S. carteri* in BC cells, further study is conducting to advance analyze the molecular mechanism of this extract inhibiting cell survival, cell migration, as well as cell proliferation. We also want to further isolate the active compounds from this extract. 

In conclusion, our data indicate a potential anti-cancer activity of ethanol extract of *S. carteri* in breast cancer cells. This research is also expected to become a consideration in escalating acquaintance of marine sponge from Indonesia. 

## Funding Statement

This OCEAN project is supported by Competence Research Grant from Universitas Padjadjaran for MHB (no.2476/UN6.C/LT/2018) and a research grant (3670/UN6.C/LT/2018) by the Ministry of Research, Technology, and Higher Education of the Republic of Indonesia.

## Statement conflict of Interest

No potential conflict of interest was reported by the authors.
